# Long term outcomes of nonmyeloablative allogeneic stem cell transplantation with TSEB TLI and ATG for Mycosis Fungoides and Sezary Syndrome

**DOI:** 10.1038/s41409-024-02236-z

**Published:** 2024-03-12

**Authors:** S. L. Morris, B. R. Thomas, R. Palanicawandar, S. Whittaker, F. Child, M. Wain, V. Sim, R. Szydlo, S. Mangar, E. Olavarria, S. Lozano Cerrada, A. Muzamil, E. Kanfer

**Affiliations:** 1 Guys Hospital, London, UK; 2 Hammersmith Hospital, London, UK

**Keywords:** T-cell lymphoma, Radiotherapy

## Abstract

Advanced stage (IIB-IVB) Mycosis Fungoides (MF) and Sezary Syndrome (SS) have a poor prognosis with median survival <5 years. We report long-term outcomes of a non-myeloablative allogeneic stem cell transplantation regimen consisting of total skin electron beam therapy, total lymphoid irradiation and antithymocyte globulin. Our prospective cohort consisted of 41 patients with a higher proportion of MF (34MF, 7SS). Acute GVHD Grade 2 to 4 was seen in 31.7% and chronic GVHD Grade 2 to 4 in 24%. The cumulative incidence of non-relapse mortality was 9.8% at 1 year and 12.6% at 2 years. At Day +90 post-transplant 66% of patients had a complete response (CR). With a median post-transplant follow up of 5.27 years, the 5-year overall survival rate was 37.7% (MF 36.7%, SS 57.1%). The 5-year cumulative incidence of progressive disease or relapse was 52.7% in all patients but only 20.8% in those with CR at transplant compared to 70.6% in those not in CR at transplant (*p* = 0.006). Long term survival is possible in advanced MF and SS with non-myeloablative transplantation and outcomes are improved in patients with CR at transplant.

## Introduction

Mycosis Fungoides (MF) and Sezary syndrome(SS) are extranodal cutaneous T cell lymphomas (CTCL) presenting in the skin. The overall incidence is 0.75 per 100,000 [1]. MF is the most common CTCL variant; SS is a rare erythrodermic variant with leukemic involvement. A third of patients present with advanced stage disease (IIB-IVB) which has a 5-year survival of 47% for Stage IIB, 37% for Stage IVA1 and 18% for Stage IVA2/IVB [2]. New treatments have shown improvements in PFS, but duration of response remains limited and no treatment has been proven to improve OS. Allogeneic HCT has been shown to induce a graft-versus-lymphoma effect and there are now several published series showing durable responses and survival [3–10]. A recently published metanalysis of 15 manuscripts and 557 patients has shown an overall survival of 40% at 3+ years, with superior OS associated with reduced intensity conditioning (58% vs 30%) [11]. However, Allo HCT has associated toxicity which makes it a difficult therapy in older patients with significant comorbidities. The risk of infectious complications is high due to a suppressed immune system and breakdown of the skin barrier, and fatal post-transplant infections have been reported [3–10]. Graft-versus-Host Disease (GVHD) is a major complication of Allo HCT which can be further complicated in patients who have fragile skin with a tendency to infection and breakdown [12]. Our protocol for Allo HCT in MF and SS patients from 2001 to 2012 involved TBI, Fludarabine, Melphalan and Alemtuzumab. While some patients have obtained long term remission, the cumulative incidence of NRM at 1 year was very high at 26% [10]. Novel non-myeloablative conditioning regimens such as TSEBT, total lymphoid irradiation and antithymocyte globulin (TSEBT-TLI-ATG) was reported in 2012 and demonstrated a lower NRM and evidence of efficacy [13]. Using this regimen Weng et al. reported a phase 2 study of 35 patients with MF and SS showing a 5-year OS rate of 56% with a 1- and 2-year cumulative incidence of NRM of 3% and 14%, respectively [14]. We adopted the TSEB-TLI-ATG regimen in 2012 and now report our long-term results.

## Methods

In 2012, we introduced a reduced intensity conditioning regimen based on the Stanford Phase 2 protocol [13, 14]. All patients were diagnosed at our institution and data was collected prospectively on our skin tumor unit research database (NRES:07/H10712/106). Patients with advanced stage MF or SS Stage IIB to IVB, age >18 y and failure of at least one prior systemic therapy were selected for transplant. Patients had to have ECOG PS 0 or 1, and no significant comorbidity that precluded systemic therapy or transplant. Donors and recipients underwent high resolution HLA typing.

The conditioning regimen consisted of TSEBT, followed by concomitant TLI and ATG. TSEBT was delivered using a modern Stanford 6-dual field technique [15]. Patients with limited disease were prescribed the low dose schedule of 12 Gy in 8 fractions over 2 weeks (12 Gy/8 f/2w) [16]. Patients with more extensive disease and erythroderma were prescribed 24 Gy/16 f/4w or, if tolerated, the full dose 30 Gy/16 f/5w [15]. The patients were then admitted to the transplant ward 1 to 4 weeks following completion of TSEBT. There was no overlap of TSEBT and TLI/ATG. Rabbit ATG was infused at 1.5 mg/kg per day for 5 consecutive days, beginning on day -11. TLI 8 Gy in 10 fractions was administered on Day -11 to day -7, and Day -4 to Day-1 with 2 fractions delivered on Day -1. All patients received unmanipulated granulocyte colony-stimulating factor-mobilised peripheral blood hematopoietic cells on Day 0. GVHD prophylaxis consisted of cyclosporine (5 mg/kg/day from Day -3 until Day +100, then tapered to discontinuation by Day +180) and mycophenolate mofetil (30 mg/kg/day from Day +1 to Day +42, then tapered to discontinuation by Day +96).

Consensus clinical endpoints and response criteria for MF and SS were used [17]. Patient demographics, overall survival (OS) defined as date of transplant to date of death from any cause [17] and progression free survival (PFS) from the date of transplantation to disease progression or death from any cause [17]. Event free survival (EFS) was calculated from the date of transplantation to disease progression, relapse in those with prior CR, initiation of lymphoma specific therapy (excluding topical steroids) or death from any cause, whichever occurred first with only the last follow up censored [13]. Graft failure was not considered an event in EFS nor censored in either PFS or EFS. GVHD was graded by current consensus criteria [18, 19, 20].

The endpoints of OS, PFS and EFS were analyzed using the Kaplan–Meier method. The cumulative incidence of progression/relapse, NRM and GVHD were calculated using the Cox proportional hazards model in which death without relapse or progressive disease was treated as a competing event and last follow up was censored. All statistical analysis were performed using R Statistical Software (R v4.2.2)

## Results

41 patients were treated between Aug 2012 and Jan 2022. The baseline characteristics of the patients treated are shown in Table 1. 34 patients had MF and 7 had SS. The cohort was heavily pretreated with a median of 4 prior systemic therapies. Large cell transformation in the skin and or nodes was present in 59% of MF and 14% of SS patients. 6 patients with MF had stage IVB disease with visceral involvement including muscle, brain, bone, breast and lung. The single patient with brain involvement had a CR to low dose whole brain radiotherapy and temozolomide prior to transplant. Global response rates to prior therapy and post TSEB pre-transplant are shown in Table 2. Specifically 24 (59%) patients were in CR, 16 (39%) in PR and 1(2%) patient had SD post TSEBT pre-transplant. 2 patients had progressive disease in the skin only following prior systemic therapy and responded to TSEBT with one in CR and one in PR post TSEBT pre-transplant. 8 patients had TSEBT omitted because either they had extensive TSEBT in the past and were close to skin tolerance, or because previous TSEBT had been ineffective. Of the 8 patients who had TSEBT omitted 3 were in CR, 4 in PR and 1 had SD at the pre-Day 0 time point. Of the 33 patients who received TSEBT, 9 received low dose 12 Gy/8 f/2 weeks, 19 received 24 Gy/16 f/4 weeks and 5 received 30 Gy/20 f/5 weeks. All patients received TLI and ATG as per protocol.

**Table 1:** Patient Characteristics.

Characteristics	*N*
Median age (y)	51 (range 21 to 69)
*Sex*	
Male	30 (73%)
Female	11 (27%)
*Diagnosis and Stage*	
Mycosis Fungoides	34
Sex M: F	23: 11
Median Age at Diagnosis	43 (range 10 to 64)
Median Age at Transplant	50 (range 21 to 69)
Stage at Diagnosis	1 A = 1, IB = 16, IIB = 10, IIIA = 2, IVA2 = 4, IVB = 1
Stage at Transplant	IIB = 9, IIIA = 3, IIIB = 1, IVA2 = 15, IVB = 6
Large Cell Transformation	20
Skin only	10
Node only	5
Skin and Node	5
Sezary Syndrome	7
Sex M: F	7: 0
Median Age at Diagnosis	55 (range 32 to 66)
Median Age at Transplant	56 (range 41 to 67)
Stage at Diagnosis	IVA1 = 4, IVA2 = 3
Stage at Transplant	IVA1 = 1, IVA2 = 6
Large Cell Transformation	1
Node only	1
Median Number of prior systemic treatments	4 (range 1 to 6)
*Time from Diagnosis to Transplant*	
<24 months	13 (32%)
> 24 months	28 (68%)
*Donor*	
MUD	27 (MF = 21, SS = 6)
SIB	11 (MF = 10, SS = 1)
HAPLO	3 (MF = 3, SS = 0)

**Table 2:** Global Response rates.

Global Response	Post prior therapy	Post TSEB pre D0	Post-Transplant D + 90
CR	8 (20%)	24 (59%)	27 (66%)
PR	28 (68%)	16 (39%)	7 (17%)
SD	3 (7%)	1 (2%)	1 (2%)
PD	2^a^ (5%)	0	6^b^ (15%)

^a^2 patients had skin only PD prior to TSEB.

^b^4 Alive with PD and 2 died with PD prior to D + 90.

27 MF/SS patients had a matched unrelated donor (MUD), 11 a sibling (SIB) matched donor and 3 a Haplotype donor. (See Table 1.)

The median donor CD34+cell dose was 6.41 × 10^6^/Kg (range 2.44–9.98), and the median donor CD3+ cell dose was 21.3 × 10^7^/Kg (range 7.9–98.9). Between Day +60 and Day +100, 23 of 37 (62%) evaluable patients had full donor chimerism. 4 patients had graft rejection/failure, 2 of whom had a second transplant and 2 died of progressive disease. Of the 2 who had a second transplant, 1 is alive in CR and one is alive with SD.

Two patients subsequently lost their graft and recovered autologous haematopoiesis, while 3 others converted to full donor chimerism, in 2 cases following donor lymphocyte therapy (DLI). Ten patients remained mixed chimeras.

10 patients received DLI at a median of 113 days post-transplant (range 27 to 432 days). 4 patient received a second DLI. The reasons for DLI were relapse in 6 patients, relapse and low chimerism in 1 patient and low chimerism in 3 patients. 4 patients went into CR following DLI. Of these 4 patients 3 remain alive in CR and 1 remains alive with disease on treatment.

All patients received their hematopoietic cell infusions as an inpatient. The median length of inpatient stay was 36 days (range 24–152 days). Post transplant toxicity and complications are shown in Table 3.

**Table 3:** Post Transplant toxicity and complications.

Post-Transplant outcome	Value
Follow up	Median 5.27 years (range 0.16 to 9.68)
Re admission within 100 days	9/37 (24%)^a^
Hospital stay after re admission	10 days (range 4–39 days)
Cause of admission	
• GVHD • Non neutropenic fever • Other	5 3 1
Post-Transplant cytopenia’s <100 days	
• Neutropenia (<0.5 × 10^9^/l) • Thrombocytopenia (<20 × 10^9^/l)	33/41, median recovery D + 17 (range 6–28 13/41, median recovery D + 10 (range 8–21)
CMV in first 100 days	10 (24.4%)
• Reactivation • Organ disease	8 (19.5%) 4 (9.8%)
Acute GVHD	18 (44%)
• Severe Grade 2 or higher • Site	13 (31.7%) Skin = 11, eyes = 1, liver = 3, lungs = 2, GI = 7, oral 1, More than 1 site = 4
Chronic GVHD	16 (39%)
• Severe Grade 2 of higher • Site	4 (10%) Skin = 11, eyes = 5, oral = 2, GI = 4, lung 2 More than 1 site = 8
Cause of Death (*n* = 22)	
• Disease Progression • Acute GVHD • Chronic GVHD • Colorectal cancer • Pneumonia	13 4 (3 in CR and 1 in PR) 3 (all in CR) 1 (in CR) 1 (in CR)

^a^4 patients died during transplant admission.

### Graft versus Host disease

**Fig. 1:**
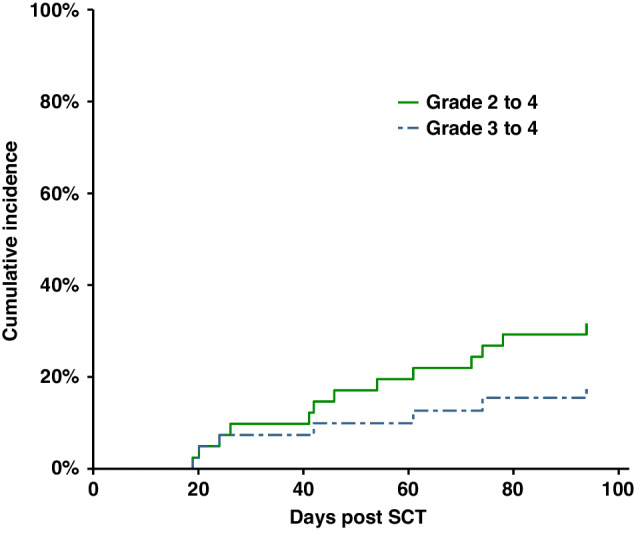
Cumulative incidence of acute GVHD.

18 (44%) patients developed Acute GVHD. The sites involved were skin (*n* = 11), eyes (*n* = 1), liver (*n* = 3), lungs (*n* = 2), gastrointestinal (*n* = 7) and oral (*n* = 1), with 4 patients having more than one site involved. Acute GVHD was Grade 2 or higher in 13 (31.7%) patients (G1 = 5, G2 = 6, G3 = 2, G4 = 5). The cumulative incidence of GVHD is shown in Fig. 1. 4 patients died related to complications of acute GVHD. 2 had SS and died of acute skin GVHD at 3.1 and 2.6 months post-transplant. 2 had MF and died of acute skin GVHD at 7.5 months and acute lung GVHD at 4.7 months.

16 (39%) patients developed chronic GVHD. The sites involved were skin (*n* = 11), eyes (*n* = 5), oral (*n* = 2), GI (*n* = 4), lung (*n* = 2) with 8 patients having more than 1 site involved. Chronic GVHD was Grade 2 or higher in 10 (24%) patients (G1 = 6, G2 = 6, G3 = 1, G4 = 3). 3 patients died of complications related to late GVHD. All three patients had MF and died in CR. One patient died at 16.4 months of chronic GVHD complications affecting GI, skin and liver. One patient died at 31.4 months of lung and liver chronic GVHD complications. One patient died at 42.6 months of skin, GI and lung chronic GVHD complications.

2 patients developed Grade 2 skin GVHD, 2.5 months and 18 months following DLI.

### Clinical response and Survival

The median follow up post-transplant of all patients was 5.27 years (range 0.16 to 9.68 years). At D + 90 post-transplant 27 (66%) patients were in CR, 7 (17%) in PR, 1 (2%) had SD and 6 (15%) had PD. Of the 6 patients who had PD, 2 died before D + 90. Of these 2 patients one had stage IVB MF and progressed with liver metastases and the other had SS (stage IVA2) and progressed in the blood. Both died of sepsis complications. Of 34 patients with MF at D + 90 23 (67%) were in CR, 5 (15%) in PR, 1 (3%) had SD and 5 (15%) had PD. 7 patients had SS and at D + 90 4 (57%) were in CR, 2 (29%) in PR and 1 (14%) had PD.

**Fig. 2:**
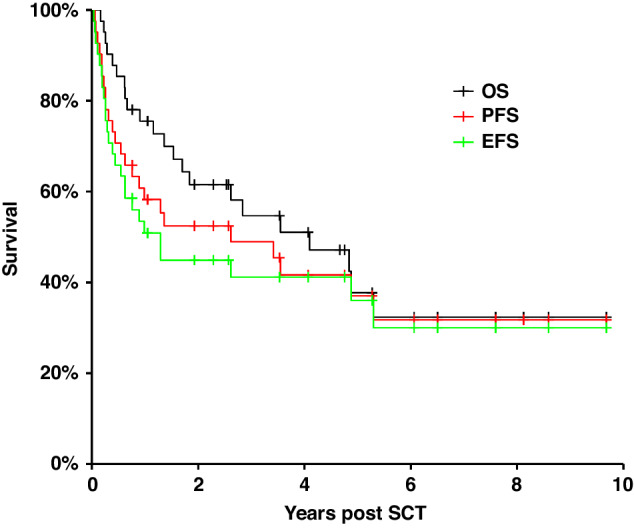
Survival post-transplant. Kaplan–Meier estimates of OS (black line), PFS (red line). EFS (green line).

The OS, PFS and EFS results are shown in Fig. 2. Median OS was 4.09 years with 2-year OS of 61.5% and 5-year OS of 37.7%. Median PFS was 2.62 years with 2-year PFS of 52.5% and 5-year PFS of 37.1%. Median EFS was 1.29 years with 2-year EFS of 45% and 5 year EFS of 36.1%.

The median OS in MF patients was 4.09 years and not reached in the SS patients. The 5-year OS in MF was 36.7% and SS 57.1% (*p* = 0.70 NS). The median survival in patients <60 y was 3.55 years compared to 4.88 years >60 y (*p* = 0.98 NS) There was no significant difference in survival by Stage. The median OS in patients with large cell transformation (LCT) was 2.83 years compared to 5.3 years in patients with no LCT but this was not significant (*p* = 0.17). The median OS by transplant type was 4.84 years for MUD, 3.55 years for SIB and 0.66 years for Haplotype-matched. Comparing the OS of the MUD/SIB patients versus the Haplotype-matched patients was significant but should be interpreted with caution given the small number of Haplotype-matched transplants (*n* = 3) (*p* = 0.03). Importantly, the median OS in patients in CR at transplant D0 was 4.88 years compared to 1.53 years if not in CR, but did not reach significance (*p* = 0.054). The median PFS in patients in CR at transplant D0 was 4.88 years compared to 0.62 years if not in CR which did reach significant (*p* = 0.02) We found no other significant findings on univariate analysis, full details are available in the Supplementary Table.

**Fig. 3:**
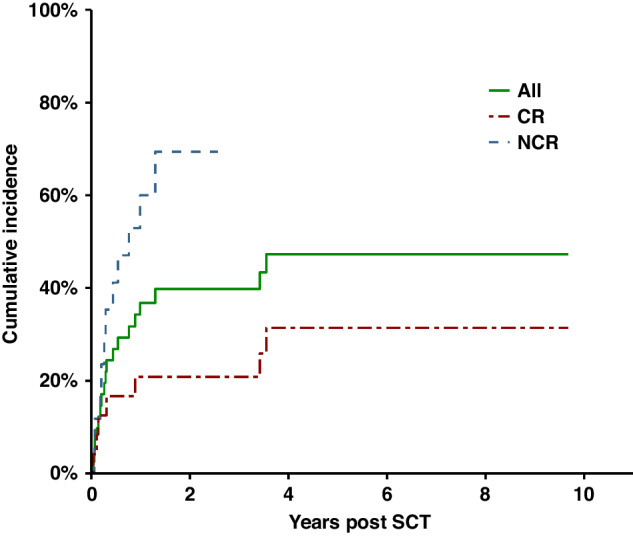
Cumulative incidence of progressive disease/relapse. Cumulative incidence of progressive disease/relapse in all, CR at D0 and NCR at D0 patients.

The cumulative incidence of PD/R post-transplant is shown in Fig. 3. The 5-year cumulative incidence was 52.7%. The cumulative incidence of PD/R in those with CR to prior therapy was 37.5% compared to 51.9% in those not in CR (Grays test *p* = 0.67 NS). The cumulative incidence of PD/R in those with CR post TSEB at D0 was 20.8% compared to 70.6% in those not in CR which was significantly different (Grays test *p* = 0.006) (See Fig. 3).

18 patients developed disease progression or relapse post-transplant (PD/R). 9 patients progressed in the skin, 1 in the blood, 4 had nodal progression and 4 had visceral progression. At last follow up 3 of these patients are alive, 2 in CR and 1 with active disease on chemotherapy. Of the CR patients, one went into CR following DLI and remains in CR at last follow-up 8.12 years post-transplant. The other patient had no donor for DLI and was treated with Brentuximab with further PD and then liposomal doxorubicin achieving a CR. This patient subsequently had a second transplant with Flu/Mel/Alemtuzumab conditioning and remains in CR at last follow-up 2.53 years post-first transplant.

**Fig. 4:**
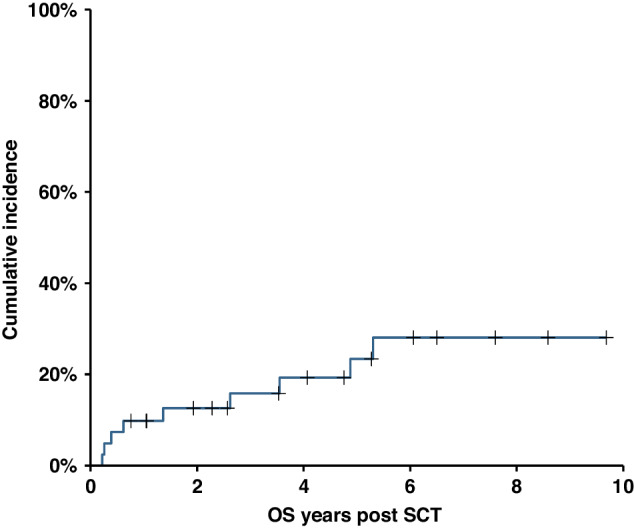
Cumulative incidence of Nn-Relapse Mortality.

At last follow-up 17 patients are alive in CR, 2 patients are alive with disease and 22 patients have died. Of the 22 deaths, 13 were due to disease progression and 9 deaths were due to non-relapse/progression. These consisted of 4 patients who died due to acute GVHD at 2.6 months, 3.1 months, 4.7 months and 7.5 months post-transplant with 3 patients in CR and one patient in PR at the time of death. 3 patients died due to chronic GVHD at 16.4 months, 31.4 months and 42.6 months post-transplant with all 3 patients in CR at the time of death. 1 patient died of pneumonia during the COVID pandemic at 5.3 years post-transplant in CR with chronic ocular GVHD. 1 patient died of metastatic colorectal cancer at 4.88 years post-transplant in CR. The cumulative incidence of NRM is shown in Fig. 4. The 1-year NRM was 9.8%, 2year NRM 12.6% and 5-year NRM 23.4%.

## Discussion

The TSEBT-TLI-ATG conditioning regimen and allo-SCT for advanced MF and SS for our patients has shown a high response rate (CR 66%) and durable remissions with a 5-year OS of 37.7%. Whilst response rates are comparable to Weng et al. our 5-year OS of 37.7% is lower than their 5-year OS of 56% but this might reflect a higher incidence of MF in our series as our SS cohort had a 5-year OS of 57.1% [14]. A 2021 update of the EBMT outcomes of patients transplanted with older conditioning regimens from 1997 to 2011 reported a similar 5-year OS of 38% but with a 2 year NRM of 28% [10]. In contrast our 2-year NRM of 12.6% is much lower and consistent with the 14% 2-year NRM reported by Weng et al. who employed a similar conditioning regimen [5, 10, 14]. A notable finding in our series is late NRM due to chronic GVHD in 2 patients with MF who died in CR of GVHD complications at 31.4 and 42.6 months post-transplant. Table 4 summarizes the demographics and results of the EBMT data, the Stanford series and our data. The Stanford series had a larger proportion of SS patients compared to MF patients in contrast to our data and the EBMT series. The difference in OS suggests that durable responses post allo-SCT may be better in leukemic disease, although PFS and EFS are similar in both series indicating that there is an effective graft versus lymphoma (GVL) effect in MF as well as SS.

**Table 4:** Comparison of Allo SCT outcomes.

	Domingo-Domenech et al. (EBMT)^a^ [10]	Weng et al.^b^ [13]	Morris et al.^b^
Number patients	113	35	41
MF:SS	77:36	13:22	34:7
Median Age (range)	48 (21 to 72)	62 (20 to 74)	50 (21 to 69)
Stage IV	50%	80%	68%
Cumulative Incidence NRM			
1 year	26%	3%	9.8%
2 year	28%	14%	12.6%
Latest NRM	14 months	NR	42.6 months
CI acute GVHD G2-4	47%	16%	31.7%
CI chronic GVHD G2-4	48%	32%	24%
5 year OS	38%	56%	37.7%
5 year PFS	26%	41%	37.1%
5 year EFS	NR	26%	36.1%

^a^multiple different conditioning regimens 1997 to 2011.

^b^identical conditioning regimen (TSEBt/TLI/ATG) 2011 to 2022.

Weng et al. also reported a significant difference in the cumulative incidence of PD/R in patients with molecular remission of 9% versus 87%. They also found a difference in PD/R in patients with clinical CR with or without molecular minimal residual disease of 13% versus 75%. We also observed a significant difference in the cumulative incidence of PD/R in patients with CR at Day 0 of 20.8% compared to 70.6% for those not in CR. In addition we observed an improvement in median OS for patients in CR at Day 0 of 4.88 years compared to 1.53 years (*p* = 0.054).

A recent update to the French propensity score matched controlled study comparing allogeneic HSCT in 55 patients to non-allogeneic HSCT in 44 patients with advanced MF and SS reported a significant improvement in PFS, but this series has only a short median follow up of 12.6 months. They report a one-year NRM of 8.5% which is comparable to our findings. This study used a variety of conditioning regimens including Fludarabine, Melphalan or Busulphan. Seven patients had a haplo donor and received conditioning with Thiotepa, Fludarabine, Busulphan and post-transplant cyclophosphamide. Unfortunately there is no detail on the outcomes of those patients who received a haplo-identical donor. Most relapses in this study were in the skin suggesting the presence of recipient derived skin resident memory tumor cells persisting after HSCT, and the authors suggest this may have been decreased by the use of TSEB as part of the transplant conditioning [21].

New therapies for CTCL have been approved over the last decade including brentuximab and mogamulizumab. Six patients in our series had stage IVB disease with visceral metastases and we would previously not have considered a transplant. Three of these six patients received brentuximab with CR in 1 patient and PR in 2 patients prior to transplant. Of these patients, two are alive in CR at 6.5 and 4.07 years, and one died of GVHD in CR at 2.62 years. Only one patient received Mogamulizumab in our series for Stage IVA2 Sezary Syndrome with a PR at D0 and subsequent CR post-transplant and remains in CR at 2 years post-transplant.

Two thirds of our patients received a matched unrelated donor which is the same proportion as in the Stanford study. Three patients received a Haplotype-matched transplant. We found no difference in survival between the MUD and SIB donors. Whilst there was a significant difference when comparing the MUD/SIB to the Haplotype-matched transplants, this should be treated cautiously as only 3 patients had a Haplotype-matched transplant. All three did not achieve a CR at transplant Day 0, and all died of disease progression.

Whilst the UK consensus guidelines recommends allo-SCT as third line for advanced MF (stage IIB to IVB), and as second line for SS (stage IVA1-IVA2) [22], the decision to transplant advanced patients remains challenging due to the variable prognosis especially for stage IIB-III MF patients. Improved prognostic markers are needed to help identify patients who need transplants. It is clear from our experience and others that patients are more likely to benefit when there is minimal disease or the patient is in clinical/molecular remission at the time of transplant. The decision to transplant should be made by an experienced team [22].

## Conclusion

In our experience the TSEBT-TLI-ATG conditioning regimen for allo-SCT in advanced MF and SS is associated with long-term survival consistent with the findings of Weng et al. [14] and provides evidence for an important GVL effect in MF as well as SS. We report a lower NRM than our previous conditioning protocol. Future studies should try to identify which patients should be considered for transplant and to determine if outcomes improve with the approval of new therapies.

### Supplementary information

SupplementaryTable

## Data Availability

We are happy to make the datasets generated and analysed during this study anonymised and available from the corresponding author on reasonable request.
