# Supplementary material for: Long term outcomes of nonmyeloablative allogeneic stem cell transplantation with TSEB TLI and ATG for Mycosis Fungoides and Sezary Syndrome
Source: Bone Marrow Transplant. 2024 Mar 12;59(6):874–9. doi: 10.1038/s41409-024-02236-z (PMC11161400; doi:10.1038/s41409-024-02236-z)
Supplement: Supplementary file 1 — SupplementaryTable [file 41409_2024_2236_MOESM1_ESM.docx]

**Supplementary table : Univariate analysis by risk factor**

|  |  | **OS** |  |  | **PFS** |  |  |
| --- | --- | --- | --- | --- | --- | --- | --- |
|  |  | n=41 |  |  | n=41 |  |  |
|  |  | **Median**  **(years)** | **HR (95% CI)** | **p-**  **value** | **Median (years)** | **HR (95% CI)** | **p-**  **value** |
|  |  |  |  |  |  |  |  |
| **Subtype** |  |  |  |  |  |  |  |
|  | **MF (ref)** | 4.09 | - |  | 3.42 | - |  |
|  | **SS** | NR | 1.27  (0.37-4.32) | 0.7 | 0.76 | 1.36  (0.46-4.02) | 0.6 |
| **LCT** |  |  |  |  |  |  |  |
|  | **No (ref)** | 5.3 | - |  | 3.55 | - |  |
|  | **Yes** | 2.83 | 1.81  (0.76-4.29) | 0.17 | 1.36 | 1.54  (0.68-3.47) | 0.6 |
| **Stage at Diagnosis** |  |  |  |  |  |  |  |
|  | **IA (ref)** | 2.62 | - |  | 2.62 | - |  |
|  | **IB** | 1.53 | 0.72  (0.09- 5.74) | 0.8 | 1.09 | 0.97  (0.12- 7.64) | >0.9 |
|  | **IIB** | 2.83 | 0.54  (0.06- 4.54) | 0.6 | 2.21 | 0.72  (0.09- 6.04) | 0.8 |
|  | **IIIA** | NR | 0  (0.00- Inf) | >0.9 | NR | 0  (0.00- Inf) | >0.9 |
|  | **IVA1** | NR | 0.25  (0.02- 4.05) | 0.3 | 0.76 | 0.58  (0.05- 6.47) | 0.7 |
|  | **IVA2** | 3.55 | 0.58  (0.06- 5.34) | 0.6 | 3.55 | 0.63  (0.07- 5.77) | 0.7 |
|  | **IVB** | NR | 0  (0.00- Inf) | >0.9 | NR | 0  (0.00-Inf) | >0.9 |
| **Stage at diagnosis** |  |  |  |  |  |  |  |
|  | **Early (IA-IIA) [ref]** | 2.62 | - |  | 1.3 | - |  |
|  | **Late (IIB-IVB)** | 4.84 | 0.56  (0.24-1.31) | 0.2 | 5.3 | 0.56  (0.25-125) | 0.2 |
| **Stage pre-transplant** |  |  |  |  |  |  |  |
|  | **IIB (ref)** | 2.83 | - |  | 0.89 | - |  |
|  | **IIIA** | NR | 0  (0.00- Inf) | >0.9 | NR | 0  (0.00- Inf) | >0.9 |
|  | **IIIB** | 1.53 | 1.75  (0.21- 14.9) | 0.6 | 0.26 | 2.49  (0.29- 21.5) | 0.4 |
|  | **IVA1** | NR | 0  (0.00- Inf) | >0.9 | 0.76 | 1.2  (0.15- 9.86) | 0.9 |
|  | **IVA2** | 4.88 | 0.58(0.20- 1.64) | 0.3 | 4.88 | 0.35  (0.13- 0.99) | 0.048 |
|  | **IVB** | 2.16 | 0.88(0.24- 3.17) | 0.8 | 1.47 | 0.58  (0.16- 2.12) | 0.4 |
| **Transplant type** |  |  |  |  |  |  |  |
|  | **Haplotype (ref)** | 0.66 | - |  | 0.26 | - |  |
|  | **MUD** | 4.84 | 0.21  (0.06-0.81) | 0.024 | 2.62 | 0.19  (0.05-0.73) | 0.016 |
|  | **SIB** | 3.55 | 0.24  (0.05-1.07) | 0.061 | 3.55 | 0.16  (0.03-0.73) | 0.018 |
| **Transplant type binned** |  |  |  |  |  |  |  |
|  | **Haplotype (ref)** | 0.66 | - |  | 0.26 | - |  |
|  | **MUD/SIB** | 4.84 | 0.22  (0.06-0.81) | 0.03 | 3.42 | 0.18  (0.05-0.68) | 0.011 |
| **Age** |  |  |  |  |  |  |  |
|  | **<60 (ref)** | 3.55 | - |  | 2.62 | - |  |
|  | **≥60** | 4.88 | 0.94  (0.31-2.8) | >0.9 | 3.09 | 1.04  (0.39-2.81) | >0.9 |
| **Stage at D0 Transplant** |  |  |  |  |  |  |  |
|  | **NCR (ref)** | 1.53 | - |  | 0.62 | - |  |
|  | **CR** | 4.88 | 0.43  (0.18-1.04) | 0.054 | 4.88 | 0.37  (0.16-0.85) | 0.02 |

Abbreviations: Overall survival (OS), Progression-free survival (PFS), number (n), Hazard ratio (HR), Confidence interval (CI), Mycosis fungoides (MF), reference group (ref), Sézary Syndrome (SS), not reached (NR), Infinity (Inf), Large cell transformation (LCT), Matched unrelated donor (MUD), Sibling (SIB)
